# A Prospective Study to Evaluate the Effectiveness of Edoxaban for the Resolution of Left Atrial Thrombosis in Patients with Atrial Fibrillation

**DOI:** 10.3390/jcm11071945

**Published:** 2022-03-31

**Authors:** Giuseppe Patti, Vito Maurizio Parato, Ilaria Cavallari, Paolo Calabrò, Vincenzo Russo, Giulia Renda, Felice Gragnano, Vittorio Pengo, Antonio D’Onofrio, Massimo Grimaldi, Raffaele De Caterina

**Affiliations:** 1 Maggiore della Carità Hospital, University of Eastern Piedmont, 28100 Novara, Italy; giuseppe.patti@uniupo.it; 2 Madonna del Soccorso Hospital, 63074 San Benedetto del Tronto, Italy; maurizioparato@alice.it; 3 Campus Bio-Medico University Hospital, 00128 Roma, Italy; i.cavallari@unicampus.it; 4 Department of Translational Medical Sciences, University of Campania Luigi Vanvitelli, 80131 Naples, Italy; paolo.calabro@unicampania.it (P.C.); felice.gragnano@unicampania.it (F.G.); 5 Division of Cardiology, Azienda Ospedaliera Sant’Anna e San Sebastiano, 81100 Caserta, Italy; 6 Cardiology Unit, Department of Translational Medical Sciences, University of Campania Luigi Vanvitelli, Monaldi Hospital, 80131 Naples, Italy; v.p.russo@libero.it; 7 Division of Cardiology, G. d’Annunzio University, SS. Annunziata Hospital, 66100 Chieti, Italy; grenda@unich.it; 8 University of Padua, Policlinico Hospital, 35128 Padua, Italy; vittorio.pengo@unipd.it; 9 Cardiology Unit, Monaldi Hospital, 80131 Naples, Italy; donofrioant1@gmail.com; 10 Regional General Hospital F. Miulli, Acquaviva delle Fonti, 70021 Bari, Italy; fiatric@hotmail.com; 11 Chair and Postgraduate School of Cardiology, University of Pisa, 56126 Pisa, Italy; 12 Cardiovascular Division, Pisa University Hospital, 56124 Pisa, Italy; 13 Fondazione VillaSerena per la Ricerca, 65013 Città Sant’Angelo, Italy

**Keywords:** atrial fibrillation, left atrium, left atrial appendage, thrombosis, vitamin K antagonists, non-vitamin K antagonist oral anticoagulants, edoxaban, thrombus resolution

## Abstract

Available evidence on left atrial (LA) thrombus dissolution in patients with atrial fibrillation (AF) largely refers to the use of vitamin K antagonist oral anticoagulants (VKAs), showing >50% thrombus resolution over a 4-week to 12-month treatment period. Available data on non-vitamin K antagonist anticoagulants (NOACs) in this setting are limited and derive from isolated case reports or observational small-sized investigations with dabigatran, rivaroxaban or apixaban. The aim of this study was to investigate the extent of thrombus resolution with edoxaban therapy in patients with AF and LA thrombosis. We conducted a prospective, observational, open-label pilot study in seven Italian institutions. We included a total of 25 patients with non-valvular AF and LA (or left atrial appendage (LAA)) thrombosis, documented by transesophageal echocardiography (TEE). All patients received edoxaban OD treatment (*n* = 23 on 60 mg daily; *n* = 2 on 30 mg daily) and underwent TEE examination after 4 weeks. The primary endpoint was the percentage of patients with complete thrombus resolution by TEE imaging at 4 weeks. The mean age of the study population was 68.3 ± 10.8 years with a female population of 16%. AF was permanent in all cases, with a mean arrhythmia duration of 4.3 ± 1.7 years. CHA_2_DS_2_-VASc and HAS-BLED scores were 3.2 ± 1.5 and 1.9 ± 1.1, respectively. We were able to demonstrate a complete thrombus resolution in 14 patients (56%) at 4 weeks. In patients with residual atrial thrombosis (*n* = 11), we observed a 15.4 ± 14.9% reduction in the thrombus area from baseline. As compared with patients without thrombus dissolution, those with thrombus resolution had a numerically lower-indexed LA diameter (27.9 ± 9.3 vs 34.8 ± 16.1 mm/m^2^), a smaller maximum thrombus area at baseline (45.5 ± 44.6 vs 63.9 ± 43.5 mm^2^), a higher left ventricular ejection fraction (47.4 ± 21.0% vs 38.4 ± 20.6%) and higher maximum LAA flow velocities (26.3 ± 15.2 vs 19.3 ± 10.0 cm/s). Figures on the percentage of thrombus resolution in this study are comparable to those reported in the literature for the other OACs. We conclude that, in patients with AF, the use of edoxaban is associated with a >50% resolution of atrial thrombus at 4 weeks, similar to studies using VKAs and the other NOACs (ClinicalTrials.gov identifier number: NCT034899395).

## 1. Introduction

The prevalence of left atrial (LA) thrombosis in patients with atrial fibrillation (AF) is relatively low (9.6% in a large observational cohort) [1] and depends on the risk profile of the considered population and on concomitant antithrombotic treatments. The detection of LA/left atrial appendage (LAA) is a contraindication to rhythm control strategies in patients with AF [2]. When atrial thrombosis is demonstrated, the largest available evidence on thrombus dissolution refers to the use of vitamin K antagonist oral anticoagulants (VKAs) [3,4,5,6]. However, here, the effectiveness of VKAs may be influenced by variability in the level of anticoagulant activity obtained, which may be suboptimal, especially in the induction phase.

In randomized phase III trials on patients with non-valvular AF, compared with the VKA warfarin, the non-vitamin K antagonist anticoagulants (NOACs) were overall at least non-inferior to warfarin for the incidence of the primary outcome, including stroke or peripheral embolism, with a better safety profile, mainly due to a significant reduction in intracranial bleeding [7]. Based on these data, NOACs are now the therapeutic strategy of choice, over VKAs, to prevent thromboembolic events in the large majority of patients with AF. Isolated reports and small observational investigations [6,8,9,10,11,12,13] have recently indicated that LA/LAA thrombus resolution may also be achieved with the use of NOACs, which ensure an immediate and stable anticoagulation. However, the efficacy of NOACs in patients with AF and documented atrial thrombosis is not, to date, robustly characterized. In particular, the above-mentioned reports essentially refer to dabigatran, rivaroxaban or apixaban, whereas no extensive, specific data on edoxaban have so far been published in this setting. Thus, the aim of this study was to investigate the extent of thrombus resolution with edoxaban therapy in patients with AF and LA/LAA thrombosis.

## 2. Methods

This was a non-controlled, observational, prospective, open-label pilot study, performed in seven Italian centers (“SS. Annunziata” Hospital, G. d’Annunzio University of Chieti (coordinating center); Campus Bio-Medico University Hospital of Rome; Cardiology Clinic, University of Padua; “Madonna del Soccorso” Hospital, S. Benedetto del Tronto; “Sant’Anna e San Sebastiano” Hospital, Caserta; Monaldi Hospital of Naples; and F. Miulli Regional General Hospital, Acquaviva delle Fonti). Patients with non-valvular AF and LA or LAA thrombosis documented at transesophageal echocardiography (TEE) were included.

The primary aim of the study was to document the extent of LA/LAA thrombus resolution upon edoxaban treatment in patients with AF. A secondary aim was to collect information for the planning and design of a larger investigation directly comparing edoxaban vs warfarin in the same clinical setting.

Specific inclusion criteria considered at the screening visit were the following: age ≥ 18 years; AF documented by electrocardiographic evidence (e.g., 12-lead electrocardiogram, rhythm strip, Holter monitoring, pacemaker interrogation) <30 days before the enrollment; a CHA_2_DS_2_-VASc score >1; and LA or LAA thrombosis documented at TEE imaging, performed prior to cardioversion/ablation or, in the case of a new AF episode, of undetermined duration.

Subjects with newly diagnosed AF were eligible, provided that there was evidence that AF was “non-valvular” and there was electrocardiographic evidence for AF on two occasions 24 h apart [14].

Exclusion criteria were:

- *Cardiac-related conditions:* hemodynamically significant mitral valve stenosis; mechanical or biological prosthetic heart valve (e.g., annuloplasty with or without prosthetic ring, commissurotomy and/or valvuloplasty permitted); transient AF caused by a reversible disorder (e.g., thyrotoxicosis, pulmonary embolism, recent surgery or myocardial infarction); known presence of atrial myxoma or left ventricular thrombus; active endocarditis.
- *Bleeding risk-related criteria:* active internal bleeding; history of conditions associated with increased bleeding risk, including, but not limited to: major surgical operation or trauma < 30 days before enrolment; clinically significant gastrointestinal bleeding < 6 months before enrolment; previous intracranial, intraocular, spinal, non-traumatic intra-articular bleeding; chronic hemorrhagic disorders; any neoplasm; arterio-venous malformation or aneurysm; a platelet count < 90,000/µL at the screening visit; sustained uncontrolled arterial hypertension (systolic blood pressure ≥ 180 mmHg or diastolic blood pressure ≥ 100 mmHg); severe, disabling stroke (with modified Rankin score of 4 to 5 after <3 months or any stroke < 14 days); transient ischemic attack (TIA) <3 days before enrolment.
- *Concomitant or intercurrent therapies:* treatment with any NOAC or VKA with optimal international normalized ratio (INR) control, defined as all INR values between 2 and 3 in the previous 30 days; aspirin > 160 mg daily; aspirin plus a P2Y_12_ inhibitor < 5 days before enrolment; intravenous antiplatelet drugs < 5 days before enrolment; fibrinolytic drugs < 10 days before enrolment; anticipated need for therapy with a non-steroidal anti-inflammatory agent in the next 4 weeks; treatment with a strong inhibitor of cytochrome P450 or inhibitors/inducers of P-glycoprotein, such as ritonavir, lopinavir, telaprevir, and indinavir, or planned treatment with such drugs during the study; other indications for anticoagulant therapy.
- *Other concomitant or intercurrent conditions*: hypersensitivity or intolerance to the study drug, including excipients; women of childbearing potential who did not want to adopt a contraceptive method during the study period and the next 4 weeks; breast-feeding women during the study period and over the next 4 weeks; anemia (hemoglobin < 10 g/dL) at the screening visit; known significant liver disease (e.g., acute clinical hepatitis, chronic active hepatitis, cirrhosis), or Aspartate Aminotransferase (AST) and Alanine Aminotransferase (ALT) > 2 × upper limit of normal (ULN) or total bilirubin > 1.5 × ULN; severe or end-stage renal disease (creatinine clearance, CrCL, <30 mL/min or on dialysis).

Enrolled patients received 60 mg of edoxaban once a day, with an open-label design, for at least 8 weeks (up to follow-up completion); edoxaban dose was to be, however, reduced to 30 mg daily in patients with CrCL 15–49 mL/min, body weight ≤ 60 kg or concomitant use of the following P-glycoprotein inhibitors: cyclosporine, dronedarone, erythromycin or ketoconazole. An electronic case report form was used, where a unique pseudo-anonymized code was assigned to each patient, and individual data—including baseline patients’ demographic details, comorbidities, thromboembolic risk (by CHA_2_DS_2_-VASc score), bleeding risk (by HAS-BLED score), vital signs, electrocardiographic findings, transthoracic echocardiography (TTE) and TEE parameters, laboratory test results (blood cell count, serum creatinine, AST, ALT) and medications—were collected. All patients underwent an ambulatory follow-up visit at 4 weeks, when the investigator-assessed clinical status, incidence of side effects or adverse events after enrollment, and therapeutic changes and compliance to edoxaban treatment were recorded. At that time, a TEE was performed to evaluate changes in LA/LAA thrombosis. The timing of repeat TEE after 4 weeks of edoxaban treatment was consistent with guideline recommendations and reported clinical experience [2,3,4,5,6,7,8,9,10]. With regard to the assessment of, adherence to, and persistence in edoxaban therapy, each patient was reminded to bring back any remaining tablets and boxes (used or unused) at the 4-week follow-up visit, at which the investigator recorded the number of supplied and remaining tablets. A final telephone assessment was undertaken for all patients at 8 weeks after the enrollment to monitor further adverse events. As the main goal of this exploratory study was an estimation of the magnitude of the LA/LAA thrombus resolution with edoxaban, no control group receiving warfarin was considered, providing that, for the purpose of this investigation, the magnitude of the response to warfarin was satisfactorily defined [2]. The rationale and design of the study have already been published [6]. The study has been registered on ClinicalTrials.gov with identifier number NCT034899395.

All patients signed an informed consent to participate in the study, which was conducted according to Good Clinical Practices (CPMP/ICH/135/95), Declaration of Helsinki and national regulations. The protocol was approved by Ethics Committees at each center. This was an investigator-initiated study, funded by Daiichi Sankyo Italy; its conduct was followed by an independent contract research organization (CRO: Hippocrates, Genoa, Italy).

## 3. Study Endpoints

### 3.1. Efficacy Assessment

The primary efficacy outcome was the percentage of patients after 4 weeks of edoxaban treatment with complete thrombus resolution by TEE imaging, using the following probe angulations: 0°, 45° to 60°, and 90°.

Secondary endpoints were:

- The percent variation of thrombus area at 4 weeks by TEE (probe angulations: 0°, 45° to 60°, and 90°);
- The incidence of thromboembolic events at 4 and 8 weeks (stroke/TIA/systemic embolism, assessed by a telephone interview).

### 3.2. Safety Assessment

The occurrence of bleeding events was assessed at 4 and 8 weeks (by a telephone interview). Major bleeding was defined as fatal bleeding; and/or symptomatic bleeding in a critical area or organ, such as intracranial, intraspinal, intraocular, retroperitoneal, intraarticular or pericardial bleeding, or intramuscular with compartment syndrome; and/or bleeding causing a fall in hemoglobin level of 20 g/L (1.24 mmol/L) or more, or leading to transfusion of ≥2 units of whole blood or red blood cells [15]. Any hemorrhagic complication that did not meet major bleeding criteria was categorized as minor.

### 3.3. Statistics

There was no previous study conducted with edoxaban on LA/LAA thrombosis resolution, so this was therefore intended as a pilot study. For this reason, the sample size was determined empirically according to previous case reports with other NOACs in the same clinical setting [6]. On this basis, we hypothesized that 60% of patients with thrombus dissolution after 4 weeks of edoxaban treatment and a sample size of 25 enrolled patients produced a two-sided 95% confidence interval, with range of 39–79%. The involvement of 7 sites implied an enrolment of 3–4 patients per site, to be recruited in 12 months.

Continuous data are reported as mean ± standard deviation and were compared by the Student t-test. Categorical variables are indicated as number (percentage), and proportions were compared by the chi-squared test. A two-sided *p*-value < 0.05 was considered statistically significant. All analyses were performed with the SPSS 24.0 software (IBM, Armonk, NY, USA).

## 4. Results

The main baseline characteristics in the 25 patients are indicated in Table 1 and Table 2, pertaining to demographic and echocardiographic parameters, respectively. The mean age was 68.3 ± 10.8 years with a 16% female population. AF was permanent in all cases, with a mean arrhythmia duration of 4.3 ± 1.7 years. CHA_2_DS_2_-VASc and HAS-BLED scores were 3.2 ± 1.5 and 1.9 ± 1.1, respectively. A 60 mg daily dose of edoxaban was given to 23 patients, and a 30 mg dose in the remaining 2, both having a CrCl 30–49 mL/min. In all cases, thrombosis at baseline was located in the LAA, being ovoid in 14 patients and pedunculated in 11. Maximum thrombus area was 70.2 ± 59.8 mm^2^.

The follow-up duration for TEE evaluation was 29.4 ± 4.0 days. A full therapeutic compliance with edoxaban was demonstrated in all patients. Complete thrombus resolution by TEE imaging was demonstrated in 14 patients (56%). In patients with residual atrial thrombosis (*n* = 11), a 15.4 ± 14.9% reduction in the thrombus area from baseline was observed. As compared with patients without thrombus resolution, those with thrombus resolution showed a numerically lower indexed LA diameter (27.9 ± 9.3 vs 34.8 ± 16.1 mm/m^2^), smaller maximal thrombus area at baseline (45.5 ± 44.6 vs 63.9 ± 43.5 mm^2^), higher left ventricular ejection fraction (47.4 ± 21.0% vs 38.4 ± 20.6%) and higher LAA maximum flow velocities (26.3 ± 15.2 vs 19.3 ± 10.0 cm/s) (Table 3). None of these comparisons were statistically significant, due to the low number of patients. Interestingly, age, prevalence of diabetes, CHA_2_DS_2_-VASc score, the number of LAA lobes, the spontaneous echo contrast grade and thrombus morphology at baseline were similar in patients with vs without complete thrombus resolution. With regard to clinical events at the 8-week follow-up (mean 56.2 ± 3.2 days), no patient had a stroke, TIA, systemic embolism or major bleeding. A minor bleeding (conjunctival bleeding) occurred in one patient more than 40 days after enrollment.

We performed an extended clinical follow-up on all patients, at a mean of 25 ± 13 months. Here, no stroke/TIA/systemic embolism occurred, three patients died (one from a myocardial infarction), and two patients had a major bleeding (on edoxaban treatment). Of the 11 patients with residual atrial thrombus at 4 weeks, three patients were switched to warfarin. In these, TEE, repeated after a mean of 2 months, revealed thrombus resolution. Eight patients continued edoxaban treatment. Among these, TEE was repeated in four patients after a mean of 3 months, demonstrating thrombus resolution, whereas the remaining four patients did not undergo further TEE examination and experienced no clinical events.

## 5. Discussion

This is the first study specifically focused on investigating, in a prospective fashion, the efficacy of edoxaban in resolving atrial thrombosis in patients with AF. We found that the use of this NOAC was associated with complete thrombus resolution in 56% of cases at 4 weeks.

In patients with AF, atrial paralysis and blood stasis, coupled with slow LAA flow [16] and atrial endocardial abnormalities, have a relevant role in a predisposition for atrial thrombosis [6]. In particular, the fibrillating atrium and LAA present the so-called ‘‘rough’’ endocardium, typically edematous, inflamed, fibrotic and dotted with thrombotic formations, as a result of mechanical, oxidative and inflammatory stressors [17,18]. Moreover, a higher exposure of procoagulant/proinflammatory factors on the atrial endocardium (e.g., von Willebrand factor [19] and tissue factor [18]) to the flowing blood, and reduced anticoagulant factors (e.g., thrombomodulin) [20] have been described, suggesting the coexistence of both local and systemic prothrombotic patterns. Increased platelet reactivity and impaired endogenous fibrinolysis have also been reported in AF [18]. By far the largest demonstration of the effectiveness of oral anticoagulation in patients with AF and atrial thrombosis refers to the use of VKAs; accordingly, in this setting, current guidelines indicate ≥ 3 weeks of treatment with such agents before cardioversion, to allow for the LA/LAA thrombosis resolution [2]. However, the efficacy of VKAs may be variable, being strictly related to the maintenance of the INR value within the therapeutic range and to the long time (in the order of 1–2 weeks) taken to achieve the INR therapeutic range [17]. In this regard, edoxaban, similar to other NOACs, may overcome the limitations of these VKAs because of fixed-dose efficacy, lack of interaction with food, and fast onset of action. Thus, uncertainties related to the effectiveness and safety of VKAs make room for NOACs as an appealing alternative in this setting, with mounting evidence arguing in favor of their possible utilization. This has been recently reinforced by recent data demonstrating that NOACs are able to limit fibrin accretion, thereby promoting endogenous fibrinolysis [21] and resulting in a further, specific mechanism for the resolution of already formed thrombi. Nevertheless, available evidence on NOACs in patients with atrial thrombosis derives mainly from anecdotal studies, isolated clinical cases or observational small-sized investigations with dabigatran, rivaroxaban or apixaban [6,8,9,10,11,12,22,23,24,25,26]. The use of edoxaban has only been reported in two patients with ischemic stroke [27], in whom the drug was effective in dissolving LAA thrombosis within 16 days. Notably, in the randomized ENSURE-AF trial [28] comparing edoxaban vs warfarin in AF patients undergoing cardioversion, if LA thrombosis was identified at TEE examination, the subject was not eligible for subsequent cardioversion. In this case, investigators were encouraged to continue treatment with the drug to which the subject had been randomized, and to repeat TEE after 4 weeks to assess the progress of the thrombus. However, a sub-analysis of ENSURE AF reported that those 89 patients (47 in the edoxaban group and 42 in the warfarin group) with LA thrombosis did not develop adverse cardiovascular events, although no mention of the percentages of thrombus resolution with the two treatment strategies at one month was provided [29].

**Figure 1:**
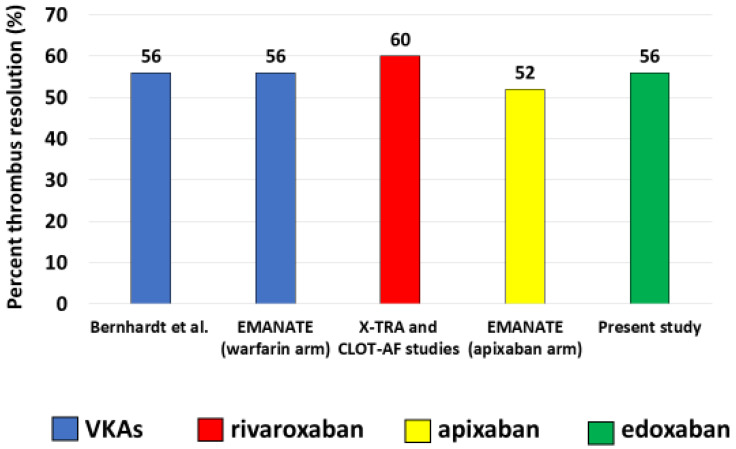
Percentage of complete left atrium/left atrial appendage thrombus resolution by transesophageal echocardiography with various oral anticoagulant approaches in observational and randomized cohort studies (as indicated) of patients with atrial fibrillation. VKAs = vitamin K antagonist oral anticoagulants.

In our population with AF and LAA thrombosis, edoxaban was given for 4 weeks and then TEE was repeated by protocol in all patients. Compliance (adherence and persistence) to the study therapy was 100%. A complete thrombus resolution was obtained in 14/25 patients (56%). Moreover, in patients without thrombus resolution, a 15 ± 15% reduction in the thrombus area from baseline was demonstrated. The above-mentioned percentage of thrombus resolution with edoxaban observed in the present investigation is comparable to that previously reported in larger studies with VKAs [4,12] or with rivaroxaban [25] and apixaban [12] (Figure 1). Notably, as compared with patients without thrombus resolution, those with thrombus resolution had a shorter AF duration, a lower left atrium size, a smaller thrombus area at baseline, a lower prevalence of heart failure, a higher left ventricular ejection fraction and increased maximum LAA flow velocities, whereas age, CHA_2_DS_2_-VASc score, prevalence of hypertension, edoxaban dose and spontaneous echo-contrast grade at the time of enrolment were similar. Previous data demonstrated a higher degree of thrombin generation in diabetic patients [30]; however, the prevalence of diabetes mellitus was here comparable in patients with and without thrombus resolution.

The present study has to be considered in light of its limitations. It is an exploratory pilot investigation enrolling a low number of patients. Its nature precluded analyses aimed at investigating independent predictors of the lack of thrombus resolution while on edoxaban treatment. By study design, no control group was included and no direct comparison between edoxaban and VKAs was performed, as our goal was also to provide data for the planning and design of a larger trial, directly comparing edoxaban vs warfarin in the same type of population. Notably, the ongoing RE-LATED AF–AFNET 7 trial has been conceived to provide a head-to-head comparison of dabigatran 150 mg BID vs phenprocoumon, with the percentage of atrial thrombus dissolution at 3–6 weeks being the main endpoint [31]. Finally, no information on thrombus resolution with edoxaban treatment longer than one month can be derived from our study.

In conclusion, our multicenter observational study on patients with AF and atrial thrombosis indicates that the use of edoxaban is associated with percentages of thrombus resolution at 4 weeks similar to those described using VKAs and the other NOACs (50% in EMANATE with apixaban, 42% with rivaroxaban in X-TRA) [12,25]. We believe these findings may impact on practice patterns and could represent the basis for a randomized comparison between NOACs and warfarin in this setting.

## Figures and Tables

**Table 1:** Main demographic/clinical features of the study population.

	*n* = 25
Age (years)	68.3 ± 10.8
Female gender	4 (16)
BMI (kg/m^2^)	28.1 ± 7.8
AF duration (years)	4.3 ± 1.7
Previous stroke	3 (12)
Heart failure	11 (44)
Arterial hypertension *	24 (96)
Diabetes mellitus **	4 (16)
COPD	6 (24)
Vascular disease	11 (44)
CHA_2_DS_2_-VASc score	3.2 ± 1.5
HAS-BLED score	1.9 ± 1.1
Baseline data	
Systolic blood pressure	127.7 ± 45.7
Diastolic blood pressure	76.4 ± 26.9
Heart rate (beats/min)	84.2 ± 30.3
Creatinine clearance (Cockroft–Gault, mL/min)	76.4 ± 20.3
Hemoglobin (g/dL)	13.5 ± 3.2
Platelet count (*n*/µL)	222,296 ± 67,310
Edoxaban dose	
60 mg	23 (92)
30 mg	2 (8)
Concomitant therapies	
Beta-blockers	19 (76)
Calcium channel blockers	9 (36)
Amiodarone	1 (4)
Digoxin	2 (8)
Propafenone	1 (4)
Hydroquinidine	1 (4)
Antiplatelet agents	6 (24)
ACE inhibitors/angiotensin receptor blockers	17 (68)
Diuretic agents	13 (52)

Data are expressed as *n* (%) or mean ± standard deviation. * Defined as current therapy with antihypertensive agent, or blood pressure values above the targets recommended by the European Society of Cardiology in patients naïve to antihypertensive agents. ** Defined as a previous definite diagnosis of diabetes mellitus by at least one of the following: fasting plasma glucose level ≥7.0 mmol/L (126 mg/dL), plasma glucose ≥11.1 mmol/L (200 mg/dL) two hours after a 75 g oral glucose load at an oral glucose tolerance test, or symptoms of hyperglycemia and an occasional plasma glucose ≥11.1 mmol/L (200 mg/dL). ACE = angiotensin converting enzyme; AF = atrial fibrillation; BMI = body mass index; COPD = chronic obstructive pulmonary disease.

**Table 2:** Echocardiographic parameters at baseline.

	*n* = 25
TTE findings at baseline	
Maximum indexed left atrial diameter (mm/m^2^)	30.6 ± 12.5
Left ventricular ejection fraction (%)	43.6 ± 20.9
Indexed left atrial volume (mL/m^2^)	44.4 ± 13.4
Indexed left ventricular end-diastolic volume (mL/m^2^)	67.7 ± 38.8
Left ventricular hypertrophy	13 (52)
Mitral regurgitation (moderate to severe)	6 (24)
Aortic valve disease (moderate to severe)	4 (16)
PASP (mmHg)	35.2 ± 12.7
TAPSE (mm)	18.3 ± 4.0
TEE findings at baseline	
Spontaneous left atrial echo contrast (at least moderate)	10 (40)
Left atrial velocity (cm/s)	23.5 ± 10.9
Site of thrombosis	
LAA	25 (100)
Other	-
Multilobes LAA	6 (24)
Thrombus characteristics	
Ovoid	14 (56)
Pedunculated	11 (44)
Thrombus measures	
Maximum thrombus area (mm^2^)	70.2 ± 59.8

Data are expressed as *n* (%) or mean ± standard deviation. LAA = left atrial appendage; PASP = pulmonary artery systolic pressure; TAPSE = tricuspid annular plane systolic excursion; TEE = transesophageal echocardiography; TTE = transthoracic echocardiography.

**Table 3:** Baseline characteristics in patients with and without thrombus resolution after 4 weeks of edoxaban therapy.

	Thrombus Resolution (*n* = 14)	No Thrombus Resolution (*n* = 11)
Age (years)	68.4 ± 10.6	67.7 ± 11.6
Female gender	3 (21)	1 (9)
BMI (kg/m^2^)	28.0 ± 9.6	28.3 ± 4.9
AF duration (years)	3.5 ± 1.4	4.5 ± 1.8
Arterial hypertension	14 (100)	10 (91)
Diabetes mellitus	2 (14)	2 (18)
Heart failure	5 (36)	6 (55)
CHA_2_DS_2_-VASc score	3.2 ± 1.3	3.4 ± 1.2
Left ventricular ejection fraction (%)	47.4 ± 21.0	38.4 ± 20.6
Maximum indexed left atrial diameter (mm/m^2^)	27.9 ± 9.3	34.8 ± 16.1
Indexed left ventricular end-diastolic volume (mL/m^2^)	62.8 ± 34.1	76.0 ± 48.5
At least moderate mitral/aortic valve disease	4 (28)	3 (27)
No. of LAA lobes	1.4 ± 0.7	1.6 ± 1.0
Spontaneous echo contrast grade	1.8 ± 0.8	2.0 ± 1.1
LAA flow velocity (cm/s)	26.3 ± 15.2	19.3 ± 10.0
Ovoid thrombus at enrollment	7 (50)	7 (64)
Maximum thrombus area at enrollment (mm^2^)	45.5 ± 44.6	63.9 ± 43.5
Edoxaban dose		
60 mg	13 (93)	10 (91)
30 mg	1 (7)	1 (9)

Data are expressed as *n* (%) or mean ± standard deviation. AF = atrial fibrillation; BMI = body mass index; LAA = left atrial appendage.

## Data Availability

Further data available upon reasonable request.

## Disclosures

G.P.	Speaker/consultant fees from Abbott, Astra Zeneca, Sanofi, Amgen, Menarini, Bayer, Pfizer, BMS, Daiichi Sankyo, PIAM, Malesci, Sigma Tau, Chiesi, Medtronic, MSD, Boehringer Ingelheim. V.M.P., P.C., V.R., F.G., V.P., A.DO., and M.G.: nothing to disclose.
I.C.	Honoraria from BMS-Pfizer and Boehringer Ingelheim.
G.R.	Speaker/consultant fees from Astra Zeneca, Bayer, BMS-Pfizer, Boehringer Ingelheim, and Daiichi Sankyo.
R.D.C.	Co-author ESC Guidelines on Atrial Fibrillation 2010–2012; Steering Committee member, National Coordinator for Italy, and co-author of APPRAISE-2, ARISTOTLE, AVERROES, ENGAGE AF-TIMI 38, and Re-DUAL PCI; fees, honoraria and research funding from Sanofi-Aventis, Boehringer Ingelheim, Bayer, BMS/Pfizer, Daiichi-Sankyo, Novartis, Merck, Portola, Roche, AstraZeneca, Menarini, Guidotti, and Milestone.
